# LEF-1 and TCF4 expression correlate inversely with survival in colorectal cancer

**DOI:** 10.1186/1479-5876-8-123

**Published:** 2010-11-22

**Authors:** Lydia Kriegl, David Horst, Jana A Reiche, Jutta Engel, Thomas Kirchner, Andreas Jung

**Affiliations:** 1 Department of Pathology, Ludwig-Maximilians-Universität (LMU), Thalkirchnerstr. 36, 80337, Munich, Germany; 2 Munich Cancer Registry (MCR) of the Munich Cancer Centre (MCC) at the Department of Medical Informatics, Biometry and Epidemiology, Ludwig-Maximilians-Universität (LMU), University Hospital Großhadern, Marchioninistraße 15, 81377 Munich, Germany; 3 Dana-Farber Cancer Institute, Boston, USA

## Abstract

**Background:**

Most colorectal carcinomas are driven by an activation of the canonical Wnt signalling pathway, which promotes the expression of multiple target genes mediating proliferation inavasion and invasion. Upon activation of the Wnt signalling pathway its key player β-catenin translocates from the cytoplasm to the nucleus and binds to members of the T-cell factor (TCF)/lymphoid enhancer factor (LEF-1) family namely LEF-1 and TCF4 which are central mediators of transcription. In this study we investigated the expression of β-Catenin, LEF1 and TCF4 in colorectal carcinomas and their prognostic significance.

**Methods:**

Immunohistochemical analyses of LEF-1, TCF4 and nuclear β-Catenin were done using a tissue microarray with 214 colorectal cancer specimens. The expression patterns were compared with each other and the results were correlated with clinicopathologic variables and overall survival in univariate and multivariate analysis.

**Results:**

LEF-1 expression was found in 56 (26%) and TCF4 expression in 99 (46%) of colorectal carcinomas and both were heterogenously distributed throughout the tumours. Comparing LEF-1, TCF4 and β-catenin expression patterns we found no correlation. In univariate analysis, TCF4 expression turned out to be a negative prognostic factor being associated with shorter overall survival (p = 0.020), whereas LEF-1 expression as well as a LEF-1/TCF4 ratio were positive prognostic factors and correlated with longer overall survival (p = 0.015 respectively p = 0.001). In multivariate analysis, LEF-1 and TCF4 expression were confirmed to be independent predictors of longer respectively shorter overall survival, when considered together with tumour stage, gender and age (risk ratio for LEF-1: 2.66; p = 0.027 risk ratio for TCF4: 2.18; p = 0.014).

**Conclusions:**

This study demonstrates different prognostic values of LEF-1 and TCF4 expression in colorectal cancer patients indicating different regulation of these transcription mediators during tumour progression. Moreover both factors may serve as new potential predictive markers in low stage colon cancer cases in advance.

## Background

Colorectal cancer is one of to the most common tumour diseases in the Western world but despite significant improvements in prevention and therapy it is one of the leading causes of cancer-related death. Dysregulation and abnormal activation of the Wnt/β-catenin signalling pathway caused by mutations of APC are decisive for the initiation as well as progression of colorectal cancer. Effects of signalling activity of β-catenin are mediated by members of the T-cell factor (TCF)/lymphoid enhancer factor (LEF-1) family. These DNA binding proteins interact with β-catenin in the nucleus and stimulate a battery of gene promoters causing proliferation, morphogenesis, epithelial-mesenchymal transition and stemness which drive neoplastic progression [1,2]. In the colorectal adenoma-carcinoma sequence genetic alterations and molecular dysregulations cause continuous stabilasation of β-cateninwhich is accompanied partly by nuclear accumulation of β-catenin in neoplastic cells. Intratumoral distribution of nuclear β-catenin is thus heterogeneous and frequently predominates at the invasive front indicating an intratumoural regulation of Wnt/β-catenin activity and its related effects [3].

Wnt/β-catenin signalling activity and its transcriptional effects might be further modulated by a variable use of the nuclear binding partners of β-catenin, namely TCF4 and LEF-1. TCF4 is the main binding partner of β-catenin in the colon and mediates transformation of colon epithelial cells upon loss of the tumour-suppressor protein APC. TCF4 has also been shown to be essential for the maintenance of the crypt stem cells of gut epithelium as TCF4 knockout mice show few differentiated villi and no proliferating crypt stem cell compartment [4]. LEF-1 on the other hand is a cell type specific transcription factor which was initially discovered in pre-T and B lymphocytes [5-7]. It belongs to the family of high mobility group (HMG) proteins which induce structural alterations in the DNA-Helix [8,9]. When overexpressed LEF-1 leads to an enhanced tumour cell invasiveness [10] and induces epithelial to mesenchymal transition [11]. Transcription of LEF-1 can be directly regulated by TCF4-β-catenin complexes [12]. As LEF-1 is not expressed in the normal colon mucosa [13], but is found in human colorectal cancer [14], a shift of β-catenin binding partners from TCF4 to LEF-1 might occur during carcinogenesis which might enable enhanced epithelial-mesenchymal transition (EMT) and malignant progression.

As systematic investigations of LEF-1 and TCF4 expression in CRC are lacking up to now, we examined the intratumoral distribution of TCF4 and LEF-1 in correlation with nuclear β-catenin using immunohistochemistry on tissue microarrays (TMA). Additionally the results were correlated with clinicopathologic variables and overall survival in univariate and multivariate analysis.

## Materials and methods

### Clinical samples

Colorectal cancer specimens from patients that underwent intentionally curative surgical resection between 1994 and 2004 at the Ludwig Maximilians-Universität München were drawn from the Institute's archives. Only colorectal adenocarcinomas with moderate differentiation (G2 according to WHO), T-categories T2 and T3 having neither nodal (N0) nor distant metastasis (M0) at the time of diagnosis were considered. To reduce surgery related effect, specimens of patients who died within 6 months after surgical resection were excluded. This resulted in a collection of tissue from 214 patients, of whom 105 (49%) died from colorectal cancer within 5 years of diagnosis. The survival data of 156 cases (73%) was censored as case follow up was discontinued or patients died of reasons other than colorectal cancer. Case characteristics are summarized in Table 1. The study complied with the requirements of the local ethics committee.

**Table 1:** Clinicopathological characteristics of the investigated colorectal cancer cases.

Variable	Number of cases	%
Gender		
Male	116	54
Female	98	46
Age, y		
< 70	120	56
≥ 70	94	44
T-category		
T2	33	15
T3	181	85
Cancer specific survival, y		
< 5	105	49
≥ 5	109	51
Censored	156	73

### Tissue microarray technique

Colorectal tissue microarrays (TMA) were constructed as described previously [15]. Briefly 5 μm sections of formalin fixed, paraffin embedded tumour samples stained with haematoxylin-eosin were used to define representative areas of viable tumour tissue. 1.0 mm needle core-biopsies were taken from corresponding areas on the paraffin-embedded tumour blocks using a tissue arraying instrument (Beecher Instruments, Sun Prarie, WI, U.S.A) and then placed in recipient paraffin array blocks at defined coordinates. To ensure that representative parts of the tumours were investigated six probes of each tumour were taken - three from central tumour areas and three from the invasive front. The cores in the paraffin block were incubated for 30 min at 37°C to improve adhesion between cores and paraffin of the recipient block.

### Immunohistochemistry

Immunohistochemical staining was done on 5 μm sections of TMA blocks. As primary antibodies, prediluted anti-β-catenin monoclonal mouse antibody (clone 14, Ventana Medical Systems), anti-LEF-1 monoclonal rabbit antibody (1:150; Cell Signaling Technology, Inc., Cat. No. 2230S, Boston, UK) and anti-TCF4 monoclonal mouse antibody (1:50; Zytomed Systems, Cat. No. 120-0036, Berlin, Germany) were used. Staining of anti-β-catenin was performed on a Ventana Benchmark XT autostainer with the XT ultraView DAB Kit (Ventana Medical Systems). For anti-LEF-1 and anti-TCF4 the sections were pre-treated for antigen retrieval by boiling in a microwave oven, twice for 15 min at 750 W in Target Retrieval Solution (Dako, Hamburg, Germany). Endogenous peroxidase was blocked by incubation in 7.5% hydrogen peroxide for 10 minutes. Detection was done using Vectastain ABC-Kit Elite Universal kits (Vector Laboratories, CA, USA) together with AEC (Zytomed Systems) as the chromogen. Finally, slides were counterstained with hematoxylin (Vector).

### Evaluation of LEF-1 and TCF4, β-Catenin immunohistochemistry

Nuclear β-catenin, LEF-1 and TCF4 staining was categorized as either positive or negative in tumour cells, while the intensity of staining was not considered. To determine the combined influence of LEF-1 and TCF4 on tumorigenesis, a LEF-1/TCF4 score was generated. Therefore, negative staining of LEF-1 or TCF4 was scored with 1 and positive staining was scored with 2. LEF-1 score was then divided by TCF4 score resulting in values ranging from 0.5 to 2. LEF-1 and TCF4 expression was moreover found in lymphocytes acting as the internal positive control. Additionally, LEF-1 and TCF4 detection was positive in the nucleus of tumour cells consistent with their function as transcription factors. Membranous β-catenin expression was not considered in the evaluation. To exclude intraobserver variability specimens were evaluated twice by an observer who had no prior knowledge of prognosis or other clinicopathological variables.

### Statistical analysis

Cross-tabulations were calculated using Fisher's exact test. Kaplan-Meier analysis was used to estimate cancer specific survival. Significance of the Kaplan-Meier statistic was tested by calculating the log-rank. Multivariate analysis was done recruiting the multivariate Cox regression model. Statistics were calculated using SPSS version 15.0 (SPSS Inc.). p-values < 0.05 were considered to be statistically significant.

## Results

### LEF-1 and TCF4 expression in colorectal cancer

**Figure 1:**
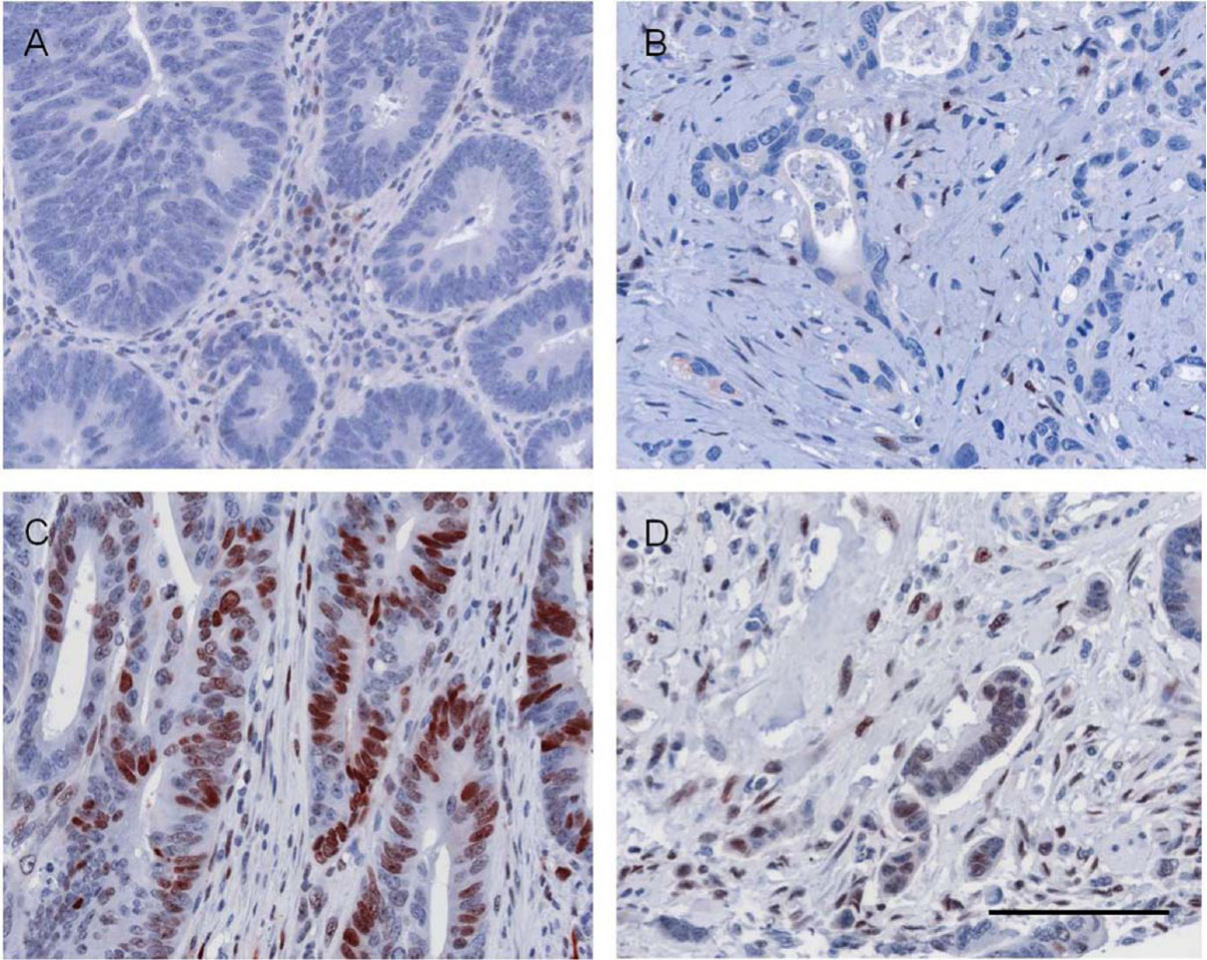
**LEF-1 expression in human colorectal cancer**. 74% of cases displayed no LEF-1 expression, neither in main tumor areas (A) nor in cells of the invasion front (B). Lymphocytes were LEF-1 positive and served as internal positive control. 26% of cases showed LEF-1 expression which could be found either only in main tumour areas (C) which occured in 16 cases or only in cells of the invasive front (D) which was seen in 5 cases or homogenously distributed throughout the tumour which was found in 35 cases. Scale bar 100 μm.

To investigate the localisation of LEF-1 and TCF4 in human colorectal cancer, we evaluated the expression of these proteins by immunostaining on tissue microarrays. LEF-1 was found to be positive in 56 cases (26%). 35 tumours displayed LEF-1 positivity both, in the tumour centre and in the front of invasion, whereas 16 cases showed LEF-1 staining only in the tumour centre. LEF-1 positivity limited to the front of invasion was found in 5 cases (Figure 1).

**Figure 2:**
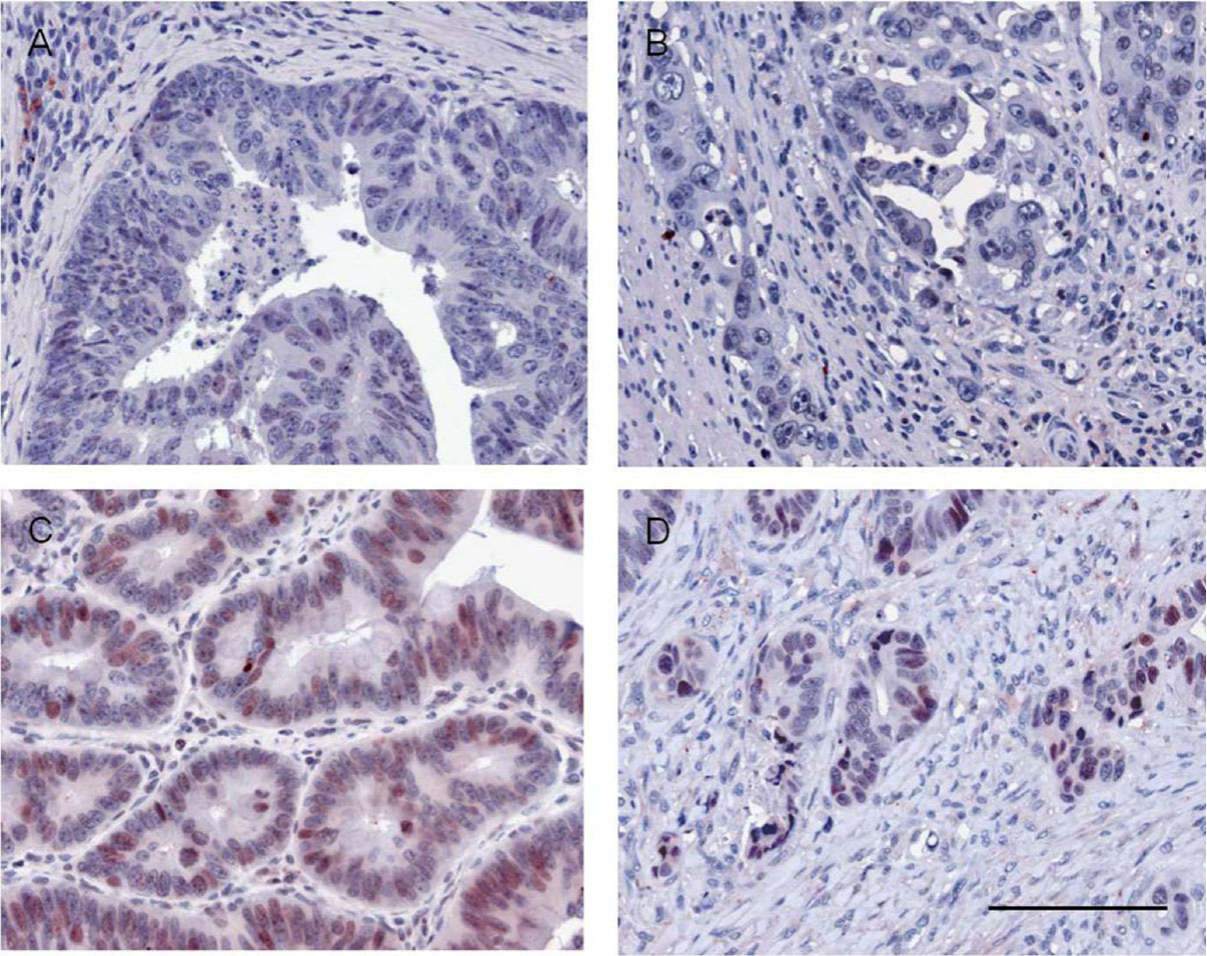
**TCF4 expression in human colorectal cancer**. 54% of cases showed no TCF4 expression, neither in main tumour areas (A) nor in cells of the invasion front (B). 46% of cases showed TCF4 expression which could be found either only in main tumour areas (C) which occurred in 28 cases or only in cells of the invasive front (D) which was seen in 6 cases or homogenously distributed throughout the tumour which was found in 65 cases. Scale bar 100 μm.

TCF4 was found positive in 99 cases (46%). 65 tumours showed TCF4 staining in the tumour centre and in the front of invasion. 28 cases exhibited TCF4 positivity only in the tumour centre and 6 cases showed TCF4 expression limited to the front of invasion (Figure 2).

### LEF-1 and TCF4 expression in colorectal cancer does not correlate with β-catenin expression

As LEF-1 and TCF4 were suggested to be important binding partners of β-catenin we next evaluated their expression in relation to nuclear β-catenin. 160 (74%) cases were positive for nuclear β-catenin staining while 54 (26%) were negative, which is in accordance to the literature [16]. 67 (42%) cases displayed nuclear β-catenin expression only in the front of invasion and 93 cases (58%) exhibited nuclear β-catenin positivity both in the tumour centre and in cells of the front of invasion. The presence and distribution of LEF-1 and TCF4 expression did not correlate with nuclear β-catenin expression (Table 2).

**Table 2:** LEF-1 and TCF4 expression are not associated with β-catenin expression

**β-Catenin positive cases**	**TCF4 positive**	**TCF4 negative**	**Total**	**β-Catenin negative cases**	**TCF4 positive**	**TCF4 negative**	**Total**
	
LEF-1 positive	19	20	39	LEF-1 positive	8	9	17
	
LEF-1 negative	55	66	121	LEF-1 negative	17	20	37
	
Total	74	86	160	Total	25	29	54

### LEF-1 and TCF4 expression in colorectal cancer correlates with patient survival

**Figure 3:**
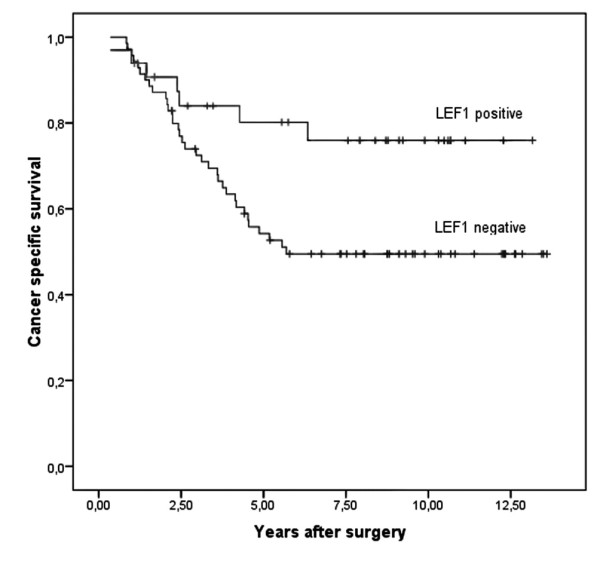
**LEF-1 expression correlates with good survival**. Kaplan-Meier plot of colorectal cancer specimens (n = 214) demonstrates significant (log-rank test) better survival with LEF-1 expression (p = 0.015).

**Figure 4:**
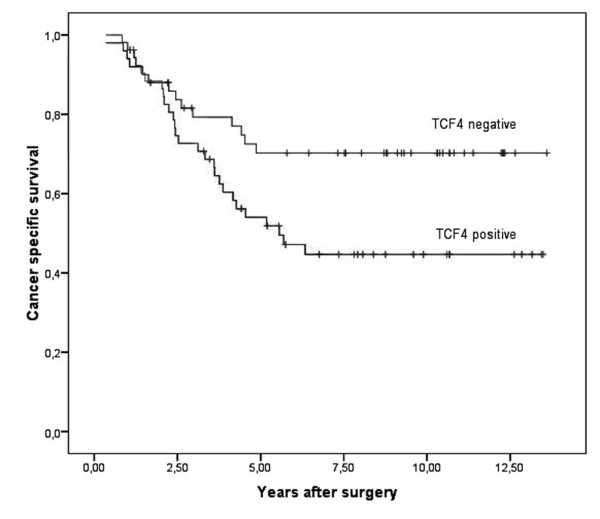
**TCF4 expression correlates with low survival**. Kaplan-Meier plot of colorectal cancer specimens (n = 214) demonstrates significant (log-rank test) worse survival with TCF4 expression (p = 0.020).

**Figure 5:**
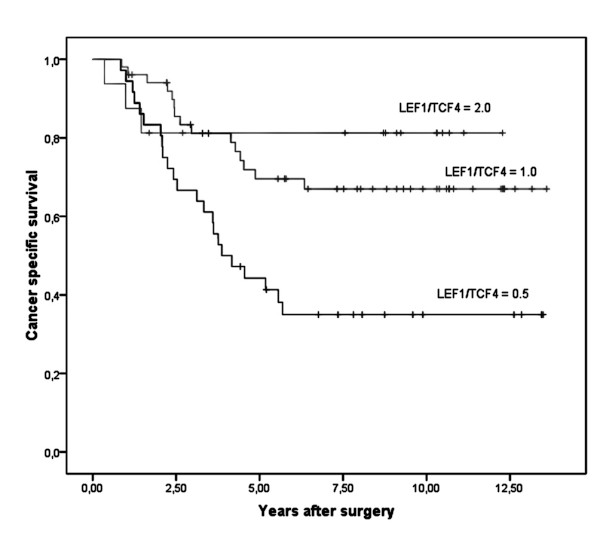
**LEF-1/TCF4 coefficient correlates with good survival**. Kaplan-Meier plot of colorectal cancer specimens (n = 214) demonstrates significant (log-rank test) better survival with high LEF-1/TCF4 ratio (p = 0.001).

Especially, we were interested to find out if LEF-1 and TCF4 expression correlates with clinicopathologic variables and with an overall clinical outcome. When comparing the LEF-1 and TCF4 status with the clinicopathological variables age, gender, and T-category of the tumour, no correlation was observed applying Fisher's exact test (Table 3 and 4). In Kaplan-Meier analyses LEF-1 positivity associated with a significant better 5- and 10 year survival of patients with colorectal cancer than LEF-1 negativity (p = 0.015; Figure 3). In contrast the presence of TCF4 expression was correlated with a significant worse 5 and 10 year survival compared to its absence (p = 0.020; Figure 4). Using a LEF-1/TCF4 ratio, we found that a high LEF-1/TCF4 coefficient correlated significantly with a better 5- and 10 year survival (p = 0.001; Figure 5).

**Table 3:** LEF-1 expression does not correlate with age, gender or T-category of the investigated colorectal cancer cases.

Variable	LEF-1 positive	LEF-1 negative	p
Gender			
Male	30	86	0.52
Female	26	72	
Age, y			
< 70	35	85	0.16
≥ 70	21	73	
T-category			
T2	20	13	
T3	43	138	0.14

**Table 4:** TCF4 expression does not correlate with age, gender or T-category of the investigated colorectal cancer cases.

Variable	TCF4 positive	TCF4 negative	p
Gender			
Male	58	58	0.15
Female	41	57	
Age, y			
< 70	55	65	0.50
≥ 70	44	50	
T-category			
T2	19	14	
T3	80	101	0.24

In a multivariate Cox regression analysis LEF-1 negativity indicated an independent relative risk of 2.66 compared to LEF-1 positivity (p = 0.027; Table 5). TCF4 expression represented an independent relative risk of 2.18 when compared to the TCF4 negative group (p = 0.014; Table 5). Age and gender were not significantly associated with outcome. Only the T-category - pT3 versus pT2 - was also significant for the outcome in multivariate analysis (p = 0.049; Table 5).

**Table 5:** Multivariate survival analysis.

Variable	Relative risk (95% confidence interval)	p
LEF-1		
Positive	1.00	
Negative	2.66 (1.11 - 6.34)	0.027
TCF4		
Negative	1.00	
Positive	2.18 (1.17 - 4.06)	0.014
Gender		
Male	1.00	
Female	0.98 (0.53 - 1.81)	0.948
Age, y		
< 70	1.00	
≥ 70	1.67 (0.91 - 3.07)	0.100
T-category		
T2	1.00	
T3	2.19 (1.28 - 6.28)	0.049

LEF-1 and TCF4 expressions in colorectal carcinoma are independent marker for patient survival.

## Discussion

The DNA binding proteins and transcription factors TCF4 and LEF-1 are partners of nuclear β-catenin and effectors of the Wnt/β-catenin signalling pathway, which is decisively involved in tumorigenesis and progression of colorectal cancer. TCF4 is present in the base of the normal colonic crypt where the TCF4/β-catenin complex controls stem cells[17]. In colorectal cancer the expression of TCF4 as well as LEF-1 has been described [14,18-20], but was not accurately evaluated and compared with nuclear β-catenin positivity.

The present study establishes that the nuclear expressions of TCF4, LEF-1 and β-catenin do not correlate with each other and that TCF4 and LEF-1 positivity is not mutually exclusive in colorectal cancer. In accordance with published literature we found nuclear β-catenin positivity in 75% of cases. In contrast LEF-1 expression was found only in 26% and TCF4 in 46% of colorectal carcinomas. Comparing LEF-1, TCF4 and β-catenin expression, there were cases without nuclear β-catenin which were positive for LEF-1, TCF4 or both factors. Additionally, other cases showed β-catenin positivity, but lacked LEF-1 and TCF4 expression. These findings suggest that activation of the Wnt signalling pathway as indicated by the presence of nuclear β-catenin staining, is not necessarily accompanied by TCF4 or LEF-1 expression. Furthermore, TCF4 and LEF-1 positivity is not restricted to β-catenin positive cases implicating the presence of Wnt-signalling-independent mechanisms, which can additionally regulate the expression of both factors in vivo.

As LEF-1 has been shown to be a target of TCF4/β-catenin [14], we speculated that tumour progression may be accompanied by a shift of β-catenin binding partners from TCF4 to LEF1 and we therefore expected to find TCF4 positivity mainly in central tumour areas and LEF-1 mainly in the front of invasion. This assumption seemed to be in accordance with studies showing that LEF-1 enhances tumour cell invasiveness [10] and induces an epithelial to mesenchymal transition [11]. However in most tumours the expression of these factors was heterogeneously distributed throughout the tumours without a discernable expression pattern. Furthermore when correlating both factors with survival we found that only TCF4 expression was associated with a significant lower overall survival, which fits with the continuous activation of the Wnt/β-catenin signalling pathway in colorectal tumorigenesis and malignant tumour progression [1,21]. In contrast LEF-1 expression and the LEF-1/TFC4 coefficient correlated with a significant better overall survival. These surprising findings suggest that TCF4 might be the main binding partner for β-catenin during development and progression of colorectal cancer whereas an enhancement of Wnt/β-catenin transcriptional activity by a switch from TCF4 to LEF-1 is unlikely. Moreover, LEF-1 expression is independent from the TCF4/β-catenin expression.

In fact, LEF-1 expression has been shown to be independently of the canonical Wnt signalling activated by the TGF-β/Smad signalling pathway [22]. Inhibition of TGFβ signalling plays a role in tumour progression of colorectal cancer [23,24] and inactivating mutations of the TGFβ pathway have been shown to cause an induction of growth arrest, differentiation and apoptosis being crucial events during the cancer progression [2,25,26]. Loss of TGF-β responsiveness promotes tumour progression in human colorectal cancers [27] and overexpression of the TGFβ inhibitor BAMBI causes colon cancer cells to form tumours that metastasize more frequently to liver and lymph nodes than control cancer cells in mural models [28]. In our study LEF-1 expression in colorectal cancer correlated with an improved patient survival. Therefore LEF-1 expression might indicate an activated TGFβ signalling which reduces tumour progression and development of metastasis.

TCF4 and LEF-1 expression was found to be heterogeneously distributed throughout the tumours, which is in support with the fact that individual tumours are organized hierarchically. Tumors display distinct sub-areas of proliferation, cell-cycle arrest, epithelial differentiation, cell adhesion and dissemination and contain different cell sup-populations like more differentiated tumor cells and tumorigenic cancer stem-like cells (CSC). CSCs are characterized by an activated Wnt/β-catenin signalling pathway [29] which is indicated by the nuclear expression of β-catenin, and EMT [30]. Dedifferentiated tumor cells with signs of EMT and nuclear expression of β-catenin which might be CSCs are found at the invasion front of colorectal cancers [31]. As LEF1 expression was found more often in main tumour areas and correlated with better survival it might indicate differentiated tumor cells without invasive or metastatic potential. In contrast TCF4 expression might indicate cells with traits of CSCs consistent with its function to maintain crypt stem cells of gut epithelium and its correlation with lower survival.

## Conclusions

In summary, we found LEF-1 expression in 26% and TCF4 in 46% of colorectal tumours. Both transcription factors were found mainly to be heterogeneously distributed throughout the tumours with expression of LEF-1 and TCF4 in cells of the invasive front in the majority of cases. Expression of LEF-1 and TCF4 did not correlate with each other or with β-catenin distribution. Furthermore we obtained evidence for a role of LEF-1 and TCF4 as independent prognostic variables of clinical outcome in colorectal tumour patients. LEF-1 expression correlated with a lower risk of death of disease and TCF4 expression correlated with a higher risk of death of disease. These results indicate different effects of the Wnt signalling pathway in vivo depending upon the nuclear binding partners of β-catenin. Moreover both factors may serve as new potential predictive markers in low stage colon cancer cases in advance.

## List of abbreviations

CSC: cancer stem cell; EMT: epithelial-mesenchymal transition; LEF-1: lymphoid enhancer factor 1; mRNA: messenger ribonuclein acid; TCF: T-cell factor; TMA: tissue microarray

## Competing interests

The authors declare that they have no competing interests.

## Authors' contributions

LK conceived the study design, carried out and coordinated immunohistochemical examinations of tumor specimens and data analysis, and drafted the manuscript. DH participated in the interpretation of data and conducted immunohistochemistry analysis. JE collected the clinical data of patients and performed statistical data analysis. AJ and TK coordinated the study and were involved in drafting the manuscript and revised it critically. All authors read and approved the final manuscript.
